# Transcatheter Aortic Valve Implantation with the Portico Valve: 2-Year Outcomes of a Multicenter, Real-World Registry

**DOI:** 10.3390/life13081785

**Published:** 2023-08-21

**Authors:** Matthaios Didagelos, Vlasis Ninios, Charalampos Kakderis, Lampros Lakkas, Antonios Kouparanis, Dimitrios Nikas, Katerina K. Naka, Aidonis Rammos, Thomas Zegkos, Vasileios Kamperidis, Ilias Ninios, Sotirios Evangelou, Dimitrios G. Tsalikakis, Lampros Michalis, Antonios Ziakas

**Affiliations:** 1 1st Cardiology Department, AHEPA University General Hospital, 54636 Thessaloniki, Greece; 2 2nd Cardiology Department, Interbalkan Medical Center, 55535 Thessaloniki, Greece; 3 2nd Cardiology Department, University Hospital of Ioannina, 45500 Ioannina, Greece; 4 Department of Informatics and Telecommunications Engineering, University of Western Macedonia, 50100 Kozani, Greece

**Keywords:** TAVI, Portico, aortic stenosis, valvular disease

## Abstract

Introduction: The self-expanding, resheathable, repositionable transcatheter aortic heart valve Portico is being used successfully for transcatheter aortic valve implantation procedures (TAVI) in patients with severe aortic stenosis. The aim of this study was to evaluate outcomes at 2 years after TAVI with the Portico valve. Methods: Multicenter registry of clinical, echocardiographic and survival data from consecutive patients treated with the Portico TAVI system (Abbott, Chicago, IL, USA) in three cath labs in Northern Greece and Epirus during 2017–2020. The primary end point was all-cause mortality at 24 months. Secondary end points included procedural outcomes (efficacy and safety) and echocardiographic measurements. Results: A total of 90 patients (81 ± 6 years, 50% females, mean age 81 ± 6 years) were included in the registry. The indication for implantation was severe, symptomatic aortic stenosis (NYHA III, IV) in eighty-two (91.1%) and degeneration of a prosthetic aortic valve in eight (8.9%) patients. All patients were categorized as high surgical risk (mean Logistic Euroscore 25.9 ± 10, Euroscore II 7.7 ± 4.4 and STS score 10.8 ± 8.9). The procedure was performed transfemorally in all patients, under general anesthesia in 95.6%, under TOE guidance in 21.1%, with native valve predilatation in 46.7%, and the “resheath” option was used in 31.1% of the cases. The implantation was successful in 97.8% and there was a need for a second valve in 2.2% of the cases. Complications included permanent pacemaker implantation (16.7%), access cite complications (15.6%), arrythmias (23.3%), paravalvular leak (moderate 7.8%, severe 1.1%), acute kidney injury (7.8%), no strokes and one death during the procedure. Aortic valve peak velocity, peak and mean pressure gradients, were significantly reduced after the procedure. All-cause mortality at 1, 12 and 24 months was 4.4%, 6.7% and 7.8%, respectively. Conclusions: TAVI with the Portico system comprises an effective and safe solution for the management of severe, symptomatic aortic stenosis in high-risk surgical patients.

## 1. Introduction

Transcatheter aortic valve implantation (TAVI) is a safe alternative method for the treatment of severe aortic stenosis in patients who are at high risk for surgical replacement of the aortic valve. The Portico transcatheter aortic valve replacement system (Abbott, Chicago, IL, USA) is composed of a bioprosthetic valve mounted within a self-expanding stent. The valve consists of three bovine pericardial leaflets and a porcine pericardial sealing cuff inside of a nitinol self-expanding frame. The system is fully resheathable, if not completely released, to achieve the optimal position in order to minimize paravalvural leaks and avoid pop-outs. The Portico valve is available in four sizes, 23 mm, 25 mm, 27 mm, and 29 mm, and has been described in detail previously [1,2].

The efficacy and safety of the Portico system has been evaluated in several registries by presenting 30-day and 1-year outcomes and in one randomized study [3,4,5,6,7,8,9,10,11,12,13,14,15]. However, long-term data >1 year are scarce [13,15]. The aim of this study was to evaluate outcomes at 2 years after TAVI with the Portico system.

## 2. Materials and Methods

### 2.1. Study Design

In this multicenter, real-world registry from 3 cath labs in Northern Greece and Epirus (AHEPA University Hospital, Thessaloniki, Interbalkan Medical Center, Thessaloniki, and University Hospital of Ioannina, Ioannina, Greece), during 2017–2020, clinical, echocardiographic and survival data from consecutive patients treated with the Portico TAVI system (Abbott, Chicago, IL, USA) were collected retrospectively. Both males and females, ≥18 years old were included in the study. The indication for implantation was severe, symptomatic native aortic stenosis or degeneration of a prosthetic aortic valve. All participants were evaluated by each institution’s Heart Team and considered high risk for surgical replacement of the valve according to the European System for Cardiac Operative Risk Evaluation score (logistic EuroSCORE or EuroSCORE II), the Society of Thoracic Surgeons Predicted Risk of Operative Mortality (STS) score or other individual risk factors not depicted in the risk scores.

All patients were evaluated with echocardiography before TAVI and underwent a coronary angiogram and CT scan to evaluate the anatomy of the coronary and the peripheral arteries and the aorta, respectively. A percutaneous coronary intervention (PCI) was performed prior to the TAVI procedure when indicated.

The study was conducted in compliance with the Declaration of Helsinki and was approved by each institution’s ethics committee.

### 2.2. Endpoints

The primary endpoint was all-cause mortality at 24 months. Secondary endpoints included procedural outcomes, and echocardiographic evaluation pre and post TAVI. All events were coded according to the standardized endpoint definitions proposed by the Valve Academic Research Consortium-3 (VARC-3) [16].

### 2.3. Statistical Analysis

Continuous variables were summarized as mean ± standard deviation and categorical variables as frequency (percentage). The echocardiographic comparisons before and after the procedures were performed with the use of paired samples t-test and the survival of the participants was calculated with Kaplan–Meier method. Statistical significance was indicated by a *p* value < 0.05. All statistical analyses were performed using IBM SPSS Statistics v23 (IBM Corp., Armonk, NY, USA).

## 3. Results

### 3.1. Baseline Characteristics

A total of 90 patients (mean age 81 ± 6 years, 50% female) underwent TAVI procedure with the Portico transcatheter aortic heart valve. The indication for implantation was severe, symptomatic aortic stenosis in 82 of them (91.1%) and degeneration of a prosthetic aortic valve in 8 cases (8.9%). All procedures were performed via the transfemoral access route. The main demographic and comorbidity data are presented in Table 1.

### 3.2. Primary Endpoint

**Figure 1:**
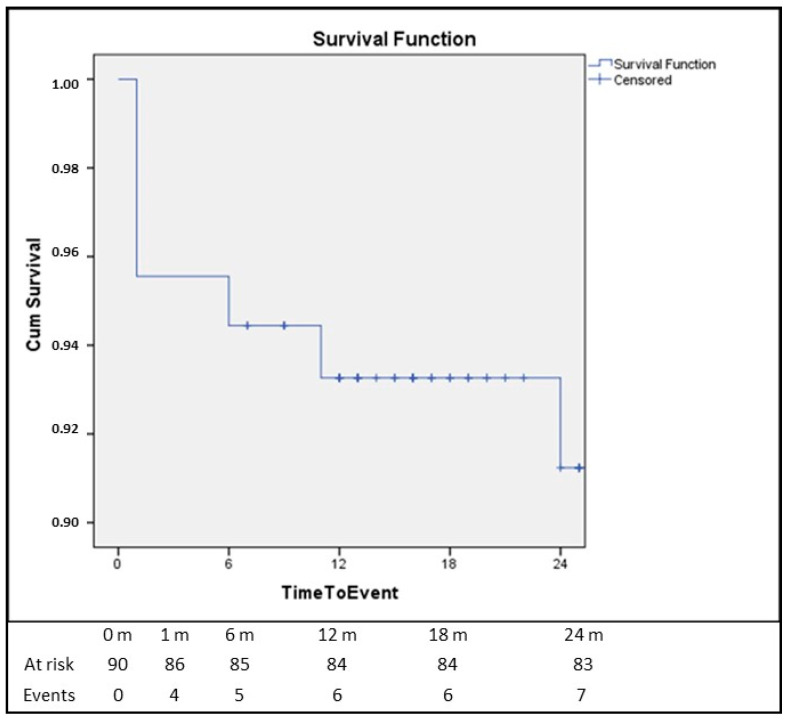
Survival at 24 months after TAVI with the Portico system.

The Kaplan–Meier survival analysis is presented in Figure 1. The patients were followed for 2 years and the survival was 95.6% at the first month after the implantation, 94.4% at 6 months, 93.3% at 12 months and 91.2% at 24 months.

### 3.3. Procedural Data

Procedural data are summarized in Table 2. General anesthesia was used in the vast majority of the procedures (95.6%) and implantations were successful in 97.8%. Transfemoral access was used in all procedures and 21.1% were additionally guided by transoesophageal echocardiogram (TOE). The valve’s resheathing capability was used in 31.1%, while the need for a second valve appeared in two procedures due to the suboptimal position of the first valve. Predilatation and postdilatation were performed in a bit less than half of the procedures (46.7% and 40%, respectively). The overlapping cusp technique was utilized in none of the patients during implantation.

### 3.4. Complications and Adverse Events

The main complications and adverse events are summarized in Table 3. Only one death occurred during the periprocedural period, and the most common complication was arrhythmias in 23.3% of the patients. Among them, 3rd degree AV block occurred in 11.1%, atrial fibrillation in 3.3% and ventricular tachycardia in 1.1%. A permanent pacemaker was implanted in 16.7% and access site complications appeared in 15.6%. More than a moderate paravalvural leak was found in 8.8% and acute kidney injury in 7.8%. No myocardial infarction, stroke or tamponade occurred. Two valves were implanted in a suboptimal position and a second valve was implanted in these two cases due to severe paravalvular leak even after post-dilatations. Major vascular complications appeared in 5.6% of the patients.

### 3.5. Echocardiographic Data

Aortic valve hemodynamics pre and post TAVI are presented in Table 4. Compared with the baseline, the aortic valve peak velocity (Vmax), the mean gradient, as well as the peak gradient were all significantly improved after the procedure (*p* < 0.001 for all of them). After the procedure, there was one severe and seven moderate paravalvular leaks (Table 3).

## 4. Discussion

In this multicenter, real-world registry of TAVI in high surgical risk patients (mean STS score 10.8%) all-cause mortality at 1, 12 and 24 months was 4.4%, 6.7% and 7.8%, respectively. One-month mortality was similar to the one been reported by previous Portico registries (2.3–4.5%). Twelve-month mortality for the Portico valve has been reported previously by four registries and one randomized study in the range of 12.1–15.8%, almost doubled compared to the results presented here. Twenty-four-month mortality for Portico is available only from the one randomized study and goes up to 22.5%, three times higher than this cohort. Three-year all-cause mortality has a rate of 35.1% and is available only by one multicenter registry (Table 5) [3,4,5,6,7,8,9,10,11,12,13,14,15].

Successful valve implantation was achieved in 97.8% of the patients, with failure of the initial attempt only in two cases because of suboptimal valve positioning, where a second valve had to be used. General anesthesia was applied in 95%, a rate much higher than that reported in more recent studies of Portico TAVI (4.9–34.3%) and although it may prolong hospitalization duration and increase complications, it is the preferred approach in the centers participating in the registry. Predilatation was used in less than half of the patients, although there is a recommendation for predilatation in all Portico implantations and the option to resheath and reposition the valve proved really helpful, as it was used in 31% of the procedures. The post-dilatation rate was high at 40%, as expected, taking into account the previous studies (15.8–43.8%) (Table 5) [3,4,5,6,7,8,9,10,11,12,13,14,15].

Serious complications like stroke and myocardial infarction did not occur in this cohort, while acute kidney injury (AKI) incidence (7.8%) was within the range reported by previous studies (1.1–7.8%). Major bleeding (VARC-3 type 2 or 3) and major vascular complication rates were also within the range of previous studies, 1.4–16.3% and 1.2–9.6%, respectively (Table 5) [3,4,5,6,7,8,9,10,11,12,13,14,15].

In this cohort, new PPM post-TAVI was required in 16.7% of the patients mainly due to third degree atrioventricular block, a rate closer to that of other registries (8.8–19.0%) and lower than that of the randomized Portico study (27.7%) (Table 5) [3,4,5,6,7,8,9,10,11,12,13,14,15].

As far as the hemodynamics have been evaluated echocardiographically, the Portico valve performed as expected by improving the peak and mean gradients across the valve and the valve area. It also improved the right ventricular and pulmonary artery hemodynamics (Table 4). A more than mild paravalvular leak remained at 8.9% of the patients, higher than the reported incidence in the literature (Table 5) [3,4,5,6,7,9,10,11,12,13,14,15].

Study limitations include the non-randomized, observational nature of this registry, the relatively small sample size, the exclusive use of the transfemoral access and the summary of data from cath labs with different experience and workloads regarding TAVI, including also the initial learning curve for all centers since their first-ever patients were also included in the analysis. However, this is the case with most other Portico studies, with only one randomized study existing so far.

## 5. Conclusions

The results of this study offer additional data that TAVI with the Portico system comprises an effective and safe solution for the management of severe, symptomatic aortic stenosis in high-risk surgical patients.

## Figures and Tables

**Table 1:** Demographic and comorbidity data of patients at baseline (*n* = 90).

Age (years)	81 ± 6
Females	45 (50%)
BMI (kg/m^2^)	28.3 ± 4.8
Logistic EuroSCORE (%)	25.9 ± 10
EuroSCORE II (%)	7.7 ± 4.4
STS score (%)	10.8 ± 8.9
Previous PCI	20 (22.2%)
Previous CABG	17 (18.9%)
Heart failure	74 (82.2%)
Arterial hypertension	79 (87.8%)
Diabetes	41 (45.6%)
Dyslipidemia	73 (81.1%)
Smoking	21 (23.3%)
Atrial fibrillation	37 (41.1%)
Any stroke	7 (7.8%)
Severe CKD (Creatinine Clearance < 50 mL/min)	15 (16.7%)
Pulmonary hypertension	20 (22.2%)
Extracardiac arteriopathy	14 (15.6%)
Pacemaker/ICD	16 (17.8%)
Previous BAV	4 (4.4%)
LBBB pre	6 (6.7%)

Data are presented in *n* (%) or mean ± standard deviation. BMI: body mass index, EuroSCORE: European System for Cardiac Operative Risk Evaluation, STS: Society of Thoracic Surgeons, PCI: percutaneous coronary intervention, CABG: coronary artery bypass grafting, CKD: chronic kidney disease, LBBB: left bundle branch block.

**Table 2:** Procedural data (*n* = 90).

Implantation success	88 (97.8%)
General anesthesia/intubation	86 (95.6%)
Conscious sedation/local	4 (4.4%)
Transfemoral access	90 (100%)
Right femoral	74 (82.2%)
Left femoral	16 (17.8%)
TOE guidance	19 (21.1%)
Pre-dilatation	42 (46.7%)
Resheath ≥ 1 times	28 (31.1%)
Portico valve size (mm)	
23	13 (14.4%)
25	23 (25.5%)
27	28 (31.1%)
29	26 (28.8%)
Suboptimal valve position	2 (2.2%)
Need for second valve	2 (2.2%)
Post-dilatation	36 (40%)
Procedure time (min)	99 ± 115
Fluoroscopy time (min)	3 ± 11
Radiation (mGy)	1703 ± 1153
Total air kerma-area product (μGy∙m^2^)	2014 ± 5348
Contrast volume (mL)	157 ± 82
New RBBB post	0
New LBBB post	14 (15.6%)

TOE: transesophageal echocardiography, RBBB: right bundle branch block, LBBB: left bundle branch block.

**Table 3:** Complications and adverse events (*n* = 90).

All-cause mortality	
30 days	4 (4.4%)
6 months	1 (5.6%)
12 months	1 (6.7%)
24 months	1 (7.8%)
Cardiovascular mortality	
30 days	3 (3.3%)
6 months	1 (4.4%)
12 months	0 (4.4%)
24 months	0 (4.4%)
Arrhythmias (any)	21 (23.3%)
3rd degree AV block	10 (11.1%)
Atrial fibrillation	3 (3.3%)
Ventricular tachycardia	1 (1.1%)
Other (PACs, PVCs, 1st or 2nd degree AV block)	7 (7.7%)
New permanent pacemaker implantation post-TAVI	15 (16.7%)
Paravalvular leak (any)	28 (31.1%)
Mild	20 (22.2%)
Moderate	7 (7.8%)
Severe	1 (1.1%)
Bleeding (VARC-3) (any)	14 (15.6%)
Type 2	10 (11.1%)
Type 3	4 (4.4%)
Vascular complications (VARC-3) (any)	14 (15.6%)
Minor	9 (10%)
Major	5 (5.6%)
AKI stage 2 or 3	7 (7.8%)
Infection	3 (3.3%)
Stroke (any)	0
Myocardial infarction	0
Tamponade	0
Coronary obstruction	0

AKI: acute kidney injury, VARC: valve academic research consortium.

**Table 4:** Valve hemodynamics pre and post TAVI (*n* = 90).

	Pre	Post	*p*
EF (%)	49 ± 13	54 ± 9	0.016
AVA echo (cm^2^)	0.7 ± 0.2	1.8 ± 0.1	<0.001
AVA index (cm^2^/m^2^)	0.4 ± 0.1	1 ± 0.1	<0.001
AV Vmax (m/s)	4.4 ± 0.6	1.9 ± 0.3	<0.001
AV peak gradient (mmHg)	80.1 ± 23.1	15 ± 4.1	<0.001
AV mean gradient (mmHg)	48.2 ± 15.6	9.1 ± 2.8	<0.001
TR Vmax (m/s)	3.1 ± 0.5	2.7 ± 0.4	<0.001
Estimated PASP (mmHg)	38.4 ± 17.3	29.2 ± 9.4	<0.001

AVA: aortic valve area, AV: aortic valve, Vmax: peak transvalvular velocity, TR: tricuspid regurgitation, PASP: pulmonary artery systolic pressure.

**Table 5:** Literature data on outcomes of TAVI with the Portico system.

	Willson et al., 2012 [3]	Manoharan et al., 2016 [4]	Perlman et al., 2017 [5]	Möllmann et al., 2017 [6] Linke et al., 2018 [8]	Denegri et al., 2018 [7]	Taramasso et al., 2018 [9]	Maisano et al., 2018 [10] Søndergaard et al., 2018 [12]	Millan-Iturbe et al., 2018 [11]	Makkar et al., 2020 [13]	Möllmann et al., 2022 [14]	Giordano et al., 2023 [15]
Study type	Two-center Registry	Multicenter Registry	Multicenter Registry	Multicenter Registry	Single center Registry	Single center Registry	Multicenter Registry	Single center Registry	Multicenter Randomized	Multicenter Registry	Multi-center Registry
Sample size	10	102	57	222	73	81	941	216	381	1001	803
STS-PROM score	8.1%	5.6%	7.7%	5.8%	4.8%	4.5%	5.8%	4.3%	6.4%	4.2%	5.2%
30-day all-cause mortality	0%	2.9%	3.5%	3.6%	2.7%	2.4%	2.7%	2.3%	4.5%	2.6%	3.9%
Implantation success	100%	98%	75.4%	99.1%	98.6%	98.7%	96%	94.4%	95.7%	97.5%	97.1%
30-day major stroke	0%	2.9%	5.3%	3.2%	2.7%	2.4%	1.6%	0.5%	1.6%	1.8%	1.4%
30-day major vascular complication	0%	5.9%	8.8%	7.2%	4.1%	1.2%	5.5%	6%	9.6%	7.3%	1.1%
30-day life-threatening or major bleeding	n/a	16.3%	12.3%	14.9%	8.2%	4.9%	3.1%	1.4%	4.8%	9.3%	1.0%
30-day permanent pacemaker	0%	9.8%	8.8%	13.5%	12%	14.2%	18.7%	15.8%	27.7%	19.0%	10.1%
Paravalvular leak ≥moderate	10%	3.8%	3.6%	5.7%	1.4%	1.2%	3.9%	3.4%	6.1%	2.1%	2%
Post-dilatation	n/a	41%	15.8%	32.7%	43.8%	n/a	43.2%	42.6%	n/a	37.6%	41.6%
12-month all-cause mortality	n/a	n/a	15.8%	13.8%	n/a	n/a	12.1%	12.3%	14.7%	n/a	n/a
24-month all-cause mortality	n/a	n/a	n/a	n/a	n/a	n/a	n/a	n/a	22.7%	n/a	n/a
36-month all-cause mor-tality	n/a	n/a	n/a	n/a	n/a	n/a	n/a	n/a	n/a	n/a	35.1%

## Data Availability

Data unavailable due to privacy.
